# Minor Surgery; or Hints on the Every-day Duties of the Surgeon

**Published:** 1843-09-02

**Authors:** 


### BIBLIOGRAPHICAL NOTICES.

*Minor Surgery; or Hints on the Every Day Duties of the Surgeon.* By Henry H. Smith, M. D., Lecturer on Minor Surgery; Fellow of the College of Physicians, etc. etc. Illustrated by Engravings. Philadelphia: Ed. Barrington & Geo. D. Haswell. 1843. 12mo. pp. 303.

This is an excellent Treatise on Bandaging, and will prove of great assistance to the student and to the country practitioner. The works of Velpeau, Gerdy, Mayor, in France, with those of several British practitioners, and the improvements in our own country, have all been liberally and judiciously used.

The first part is on the Preparation and Application of Dressings. Part second comprises the Preparation and Application of the Bandage. Mayor of Lausanne's handkerchief system is fully treated of in a separate section, and is a valuable portion of the work. Part third treats of the Apparatus for Fractures and Dislocations. The fourth and last part includes some of the Minor Surgical Operations. This portion of the work should be materially extended to warrant the title of Minor Surgery given to the book; and in another edition, which we do not doubt will be speedily called for, we hope Dr. Smith will enlarge this department, and place it on a level with the rest of the work; by so doing he will add greatly to its completeness and value. Some of his descriptions might, we think, be somewhat amplified, which would render them clearer, and more readily comprehended by the young student. At page 260 (et seq.) we have a notice of the treatment of Fractures by the Immoveable Apparatus of Seutin (which our author spells Suetin) and Velpeau. A description of the manner of application and its effects are shown by the republication of some six cases which were treated in the surgical wards of the Pennsylvania Hospital in 1838, from the Medical Examiner of 1839. The source of these cases, we think, have not been too clearly indicated by our author. No mention is made of the improvements in this important mode of treatment, and of the substitution of dextrine for starch, or other modifications, which an extended series of observations in France, Germany, Great Britian and this country, for the last six years, have induced.

At page 207 there is a description given of an excellent Apparatus for Fractured Clavicle, which Dr. Smith has erroneously attributed to Dr. Fox. The modifications are due to Dr. J. A. Washington, then the colleague of Dr. F. in the Pennsylvania Hospital. (Medical Examiner, Vol. 1, p. 108, 1838.)

We find continually through the book "the Hospital Apparatus"—"in the Hospital." By this, we presume, our author means the treatment pursued in the Pennsylvania Hospital; or the apparatus most commonly used in that institution. We think the phrase an objectionable one, and open to misconstruction. The English word *mesh* might, we think, be advantageously substituted for the French word *mèche.* We mention these as some of the blemishes we have noticed in the work, and which, we have no doubt, will be reformed in a second edition.

The book is very handsomely illustrated with a great number of excellent wood cuts. The paper and type are good. We repeat, in conclusion, our very favourable estimation of its merits.

